# Translating multimodal foundation models into oncology: Toward a future where AI directs diagnosis and therapy

**DOI:** 10.1016/j.gendis.2025.101958

**Published:** 2025-11-27

**Authors:** Kyung Chan Park, Wonbeak Yoo

**Affiliations:** Genomic Medicine Research Center, Korea Research Institute of Bioscience and Biotechnology (KRIBB), Daejeon 34141, Republic of Korea; Department of Functional Genomics, University of Science and Technology, Daejeon 34141, Republic of Korea; Genomic Medicine Research Center, Korea Research Institute of Bioscience and Biotechnology (KRIBB), Daejeon 34141, Republic of Korea

Recent developments in multimodal artificial intelligence (AI) have begun to transform how clinicians approach cancer prognosis and treatment selection. In a recent study, Xiang et al^1^ present MUSK (Multimodal Unified Self-supervised learning for Oncology), a foundation model that integrates more than 50 million whole-slide pathology images and over 1 billion oncology-related clinical text tokens. MUSK uses a unified transformer architecture to simultaneously capture morphological and semantic features, enabling the integrated image–text interpretation essential for oncology (Fig. 1A). The model was pretrained in two stages: the first stage employed masked modeling using unpaired data from each modality, while the second stage used approximately one million paired image–text samples with contrastive learning to align histologic and linguistic representations. This approach enabled robust cross-modal understanding, supporting downstream diagnostic and prognostic applications.

**Figure 1:**
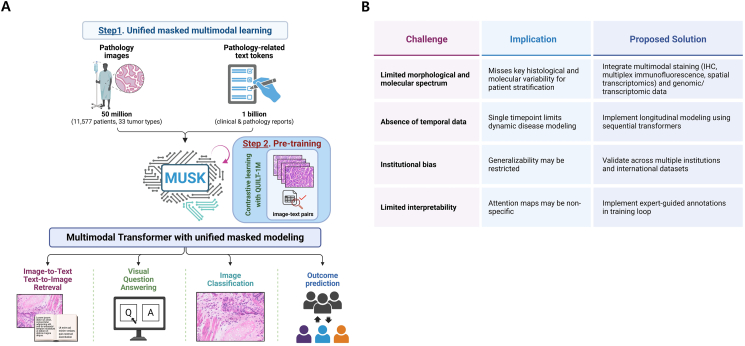
Schematic representation of the MUSK (Multimodal Transformer with Unified Masked Modeling) architecture and its potential applications in translational oncology. **(A)** The model was pretrained using a large corpus of whole-slide histopathological images and associated clinical texts derived from pathology reports, allowing integrated representation of morphologic and contextual clinical features. Pretraining was performed in two stages: an initial phase using masked modeling on unpaired data from each modality, followed by contrastive learning on ∼1 million paired image–text samples to align semantic and histologic representations. Core applications include classification of histological patterns, cross-modal retrieval between images and clinical documentation, interpretation of complex diagnostic queries, and prediction of clinically relevant endpoints such as disease recurrence, overall prognosis, and response to immune checkpoint inhibitors **(B)** This table outlines the principal limitations observed in the current implementation of the MUSK foundation model and delineates pragmatic solutions aimed at optimizing its clinical utility. Challenges include restricted morphological input due to reliance on hematoxylin-eosin staining, absence of temporally resolved patient data, and institutional sampling bias that may hinder external validity. Furthermore, a lack of integration with molecular profiles and limited granularity in interpretability mechanisms could constrain its precision in therapeutic stratification. Recommended strategies to overcome these limitations involve the adoption of multiplexed histological techniques, the incorporation of longitudinal and multi-institutional datasets, and the integration of genomic and transcriptomic layers. Expert-in-the-loop annotation frameworks may further bolster the clinical reliability of attention-based outputs.

In comparative evaluations across several cancer types, including non-small cell lung cancer, colorectal carcinoma, gastric adenocarcinoma, and melanoma, MUSK outperformed traditional staging systems and biomarker-based models. In non-small cell lung cancer, the model demonstrated refined stratification of survival risk within the same pathological stage, capturing histologic subtleties and semantic modifiers within pathology reports that may be overlooked by standard criteria. For immunotherapy response prediction, MUSK achieved an AUC of 0.77 versus 0.61 for PD-L1-based classifiers. In patients with melanoma, recurrence risk prediction reached an accuracy of 83%, with the model prioritizing regions of mitotic activity, tumor regression, and lymphovascular infiltration. Interpretability was central to the framework, with visualization tools highlighting key histologic regions and textual descriptors. These interpretive tools may aid pathologists and oncologists in understanding the model's reasoning and assessing case complexity, thereby facilitating integration into clinical decision-making workflows, including tumor board discussions and therapeutic stratification.

Compared with contemporaneous multimodal approaches, MUSK presents a more accessible and flexible architecture. M3FM, a model trained on radiologic images and structured EHR data, is optimized for lung cancer screening but is dependent on imaging modality standardization and structured clinical data input.^2^ MGCT aligns histopathology with genomic profiles for subtype prediction but requires access to next-generation sequencing, limiting its application in settings without molecular diagnostics.^3^ CPath-Omni, a patch-level computational pathology framework, demonstrates strong performance in image-based prediction tasks but remains constrained by its reliance on paired annotations and high-quality imaging infrastructure.^4^ MUSK, by contrast, operates on unpaired, routinely available hematoxylin-eosin-stained whole-slide images and unstructured clinical documentation, enhancing deployment potential in low- and middle-income countries where access to omics technologies and curated datasets is often limited. The formal peer-reviewed publication of MUSK in *Nature* (January 2025) further supports its methodological rigor and translational relevance. This distinguishes MUSK from models that remain at the preprint or prototype stage, and affirms its readiness for prospective clinical research. The study provides comprehensive documentation of the training pipeline, including dataset composition, augmentation strategies, contrastive learning objectives, and evaluation metrics across multiple institutions and tumor types.

Future directions for MUSK include the incorporation of temporal data streams to support dynamic modeling of disease progression. In longitudinal oncology care, particularly in relapsing or indolent tumors, the capacity to integrate serial pathology, follow-up imaging, and evolving treatment regimens may allow the model to identify early signs of recurrence, resistance, or therapeutic response adaptation. Such temporally resolved modeling is expected to enhance clinical utility, especially in complex treatment landscapes involving immunotherapy, targeted agents, and combined modalities. Another key area of development involves integrating histopathologic information with multi-omics data. The fusion of spatial transcriptomics, proteomics, and genomics with histologic architecture may improve biological stratification and enable mechanism-based therapeutic prediction. Existing frameworks such as iIMPACT have demonstrated that combined analysis of hematoxylin-eosin-stained slides with spatial transcriptomic data enhances spatial domain delineation and molecular subtype assignment.^5^ These approaches may be adapted to augment MUSK, allowing the model to incorporate molecular heterogeneity and cellular topography in its predictive logic.

Despite its strengths, the clinical translation of MUSK necessitates rigorous prospective validation. While retrospective analyses offer strong preliminary evidence, real-world application requires demonstration of reproducibility, robustness, and equity across diverse patient populations and clinical settings. Multicenter studies with standardized imaging protocols, harmonized reporting formats, and outcome-linked datasets will be essential for evaluating generalizability. Furthermore, assessing model performance in underrepresented populations, including minority groups, elderly patients, and rare tumor subtypes, will be crucial to ensuring ethical and inclusive deployment (Fig. 1B). To facilitate clinical adoption, MUSK's outputs must be interpretable and actionable. Visual attention maps and linguistic attributions provide a foundation for human-in-the-loop review, enabling clinicians to interrogate the model's predictions and validate findings. However, further work is needed to establish standardized interpretability benchmarks and validate whether the model's highlighted features align with expert diagnostic reasoning. Regulatory considerations will also play a role in clinical integration, as explainability, reproducibility, and auditability remain key requirements for approval of machine learning tools in clinical oncology.

Recent developments in computational oncology highlight self-supervised learning, domain adaptation, and federated training as effective strategies for model generalization without centralized data sharing. MUSK employs a self-supervised framework that enables representation learning from unannotated clinical data, enhancing adaptability across variable practice settings. Domain-specific tuning may improve robustness under differences in staining, scanner calibration, or documentation styles. Federated learning allows local model updates without transferring patient data, supporting broader deployment across institutions. Graph-based modeling offers an additional layer of interpretability by encoding cellular architecture as spatial graphs. Nodes represent nuclei or cell clusters, with edges reflecting topological proximity. This structure captures microenvironmental features such as tumor–stroma interaction and immune infiltration. Combined with transformer-based image and text embeddings, such hybrid models may improve biologically grounded prediction.

In conclusion, MUSK demonstrates the feasibility and potential of self-supervised, multimodal learning in oncology. Its ability to operate on standard histologic slides and unstructured clinical documentation—without reliance on paired inputs or advanced molecular assays—offers a scalable and interpretable approach to precision medicine. With further prospective validation, incorporation of temporal and multi-omics data, and continued attention to ethical deployment, MUSK represents a foundation for the next generation of integrative diagnostic and prognostic tools in cancer care.

## CRediT authorship contribution statement

**Kyung Chan Park:** Investigation, Methodology, Validation, Writing – review & editing. **Wonbeak Yoo:** Writing – review & editing, Writing – original draft, Project administration, Funding acquisition, Conceptualization.

## Funding

This work was supported by the 10.13039/501100003725National Research Foundation of Korea grant funded by the Korea government (MSIT) (No. RS-2024-00338524), the KRIBB Research Initiative Program (No. KGM5192531), and Seoul Clinical Laboratories (No. 2025AR01).

## Conflict of interests

All authors declared no potential conflict of interests.
